# Supplementary material for: Development of core outcome sets for clinical trials in temporomandibular disorders: A study protocol
Source: PLoS One. 2022 Apr 28;17(4):e0267722. doi: 10.1371/journal.pone.0267722 (PMC9049344; doi:10.1371/journal.pone.0267722)
Supplement: Attachment — Submitted filename: Response to Reviewers.docx [file pone.0267722.s003.docx]

Rio de Janeiro, April 04^th^ 2022

Dear Editor,

We thank very much the reviewers for the helpful suggestions regarding the manuscript titled *“***Development of Core Outcome Sets for Clinical Trials in Temporomandibular Disorders: A Study Protocol”** submitted to *Plos One.*

All requested changes have been made and highlighted in the updated version of the manuscript. Please find below specific answers addressing comments from the reviewers.

**Journal Requirements**

When submitting your revision, we need you to address these additional requirements.

1. Please ensure that your manuscript meets PLOS ONE's style requirements, including those for file naming. The PLOS ONE style templates can be found at

<https://journals.plos.org/plosone/s/file?id=wjVg/PLOSOne_formatting_sample_main_body.pdf>

and

<https://journals.plos.org/plosone/s/file?id=ba62/PLOSOne_formatting_sample_title_authors_affiliations.pdf>

2. Your ethics statement should only appear in the Methods section of your manuscript. If your ethics statement is written in any section besides the Methods, please move it to the Methods section and delete it from any other section. Please ensure that your ethics statement is included in your manuscript, as the ethics statement entered into the online submission form will not be published alongside your manuscript.

3. Please ensure that you refer to Figure 1 in your text as, if accepted, production will need this reference to link the reader to the figure.

4. Please include captions for your Supporting Information files at the end of your manuscript, and update any in-text citations to match accordingly. Please see our Supporting Information guidelines for more information: <http://journals.plos.org/plosone/s/supporting-information>.

5. Please review your reference list to ensure that it is complete and correct. If you have cited papers that have been retracted, please include the rationale for doing so in the manuscript text, or remove these references and replace them with relevant current references. Any changes to the reference list should be mentioned in the rebuttal letter that accompanies your revised manuscript. If you need to cite a retracted article, indicate the article’s retracted status in the References list and also include a citation and full reference for the retraction notice.

**Response: The manuscript has been fully revised in order to match all the journal’s requirements.**

**Additional Editor Comments:**

This is a relevant and well-written study protocol that aims to develop a core outcome sets (COS) for clinical trials in intra-articular temporomandibular disorder and masticatory muscle disorders. A few suggestions were made by the reviewers and I would like to ask the authors to revise the article.

**We highly appreciate the editor’s comments. The article has been fully revised. All comments and suggestions have been considered and all comments have been addressed.**

**Reviewer’s Comments to the Author**

**Reviewer #1:** I want to congratulate the authors on a very important well designed and mostely needed study in the field of TMD treatment.

**We highly appreciate the reviewer’s comments.**

**Reviewer #2:** The authors have developed an interesting and useful study protocol on the outcome sets for clinical trials in intra-articular TMD and masticatory muscle disorders However, I would like to make some observations before recommending your work for publication.

**We highly appreciate the reviewer’s comments.**

**Commmnet #1:**

I recommend the authors in the introduction, when discussing the articular causes of TMD, to mention a possible relationship between the systemic origin of OA and the intestinal microbiota, mentioning the following reference: doi:10.3390/nu13030716.

**Response: We appreciate the reviewer’s comment. The suggested reference has been added to the manuscript:**

11.Sanchez Romero EA, Melendez Oliva E, Alonso Perez JL, Martin Perez S, Turroni S, Marchese L, et al. Relationship between the Gut Microbiome and Osteoarthritis Pain: Review of the Literature. Nutrients. 2021;13(3). Epub 20210224. doi: 10.3390/nu13030716. PubMed PMID: 33668236; PubMed Central PMCID: PMCPMC7996179.

**Commmnet #2:**

I believe it is not necessary to include an "Objective" section at the end of the introduction.

**Response: We have removed this section, following the revoewer’s comment.**
